# Farnesyl pyrophosphate synthase promotes restenosis after vascular injury by activating small G proteins

**DOI:** 10.1016/j.jbc.2025.110861

**Published:** 2025-10-27

**Authors:** Xiaowei Liu, Lin Yang, Zhi Zhang, Yajun Liu, Wenhao Huang, Jingyuan Zhang, Qingqing Chen, Lijiang Tang, Changqing Du

**Affiliations:** 1 Department of Cardiology, Zhejiang Hospital, Hangzhou, Zhejiang, PR China; 2 Department of Geriatrics, First Affiliated Hospital of Zhejiang University, Hangzhou, Zhejiang, PR China; 3 School of Pharmaceutical Science, Zhejiang Chinese Medical University, Hangzhou, Zhejiang, PR China; 4 The Second Clinical Medical College, Zhejiang Chinese Medical University, Hangzhou, PR China

**Keywords:** farnesyl pyrophosphate synthase, restenosis, vascular injury, small G proteins, mitogen-activated protein kinase

## Abstract

This study aimed to investigate the role of farnesyl pyrophosphate synthase (FPPS) in restenosis following vascular injury. Primary vascular smooth muscle cells were transfected with or without Lenti-FPPS shRNA and subsequently stimulated with angiotensin II (10^-7^ mol/l) or transforming growth factor-beta 1 (10 ng/ml). The activities of small G proteins were assessed. An *in vivo* vascular restenosis model was established by inducing balloon injury in the rat carotid intima. Injured carotid arteries were transfected with Lenti-FPPS shRNA (6 × 10^8^ plaque-forming unit), and vascular histopathology was analyzed. Gene and protein expression levels were evaluated in both *in vivo* and *in vitro* models using quantitative RT–PCR and Western blotting, respectively. Angiotensin II or transforming growth factor-beta 1 stimulation increased the protein levels of FPPS, connective tissue growth factor, phosphorylated p38, and Jun NH2-terminal kinase, as well as the activities of small G proteins in vascular smooth muscle cells. These effects were significantly attenuated following Lenti-FPPS shRNA transfection. In the carotid artery injury model, rats exhibited luminal narrowing, extensive neointima formation, and abundant foam cells and fibrous connective tissue in the intima and media. However, these pathological changes, along with the upregulation of FPPS, connective tissue growth factor, and small G protein activities in the injured carotid arteries, were alleviated by FPPS inhibition *via* Lenti-FPPS shRNA transfection. This study demonstrates that FPPS contributes to restenosis after vascular injury, potentially by modulating the small G protein (Rac and RhoA)–Jun NH2-terminal kinase and small G protein (Rac and RhoA)–p38 mitogen-activated protein kinase signaling pathways in both *in vitro* and *in vivo* models.

Coronary heart disease (CHD) is a leading cause of morbidity and mortality worldwide, posing a significant threat to global health (1, 2). Since Grüntzig (3) performed the first percutaneous transluminal coronary angioplasty (PTCA) in 1977, PTCA has become a widely adopted treatment for CHD. Current therapeutic approaches for CHD include pharmacological therapy, interventional procedures, and coronary artery bypass grafting. Among these, percutaneous coronary intervention (PCI) remains the primary choice for managing severe angina pectoris, myocardial ischemia, and myocardial infarction due to its ability to rapidly restore blood flow with minimal trauma and a high success rate of approximately 97% (4, 5). Although interventional therapy for CHD has the clearly beneficial effects of opening the culprit blood vessel, less trauma, quick recovery, and a success rate as high as 97%, it has the disadvantage of restenosis after stenting (6, 7). Indeed, about 15% to 30% of the patients developed restenosis within 3 to 6 months after PTCA (8).

Various mechanisms have been proposed to explain restenosis, including elastic recoil, thrombosis, neointimal hyperplasia, and vascular remodeling (9, 10, 11). The fundamental principle of PTCA involves mechanical expansion, which induces localized disruption of the intima and media at the lesion site, leading to vessel dilation and restoration of blood perfusion (12). However, this mechanical intervention inevitably triggers a cascade of vascular repair responses, including the proliferation of intimal, medial, and adventitial cells, as well as fibrous tissue formation, resembling general wound healing processes (13, 14). These mechanisms are similar to those involved in general wound healing processes and are the primary cause of restenosis. In addition, platelet aggregation and thrombus reorganization at the site of mechanical expansion or stent implantation, along with the elastic recoil of the expanded vessel wall induced by mechanical stretching, can further exacerbate restenosis (15, 16). Currently, the issues of elastic recoil and thrombosis have been largely mitigated through the use of drug-eluting stents (17). However, intimal hyperplasia and vascular remodeling remain major contributors to restenosis, and developing effective strategies to prevent and control these pathological processes remains a key research focus in interventional cardiology.

Restenosis is primarily driven by neointimal proliferation, collagen synthesis, and extracellular matrix (ECM) secretion from vascular smooth muscle cells (VSMCs) (18). Therefore, identifying an effective and economical strategy to inhibit neointimal proliferation and collagen fiber synthesis could provide a fundamental solution to restenosis. Farnesyl pyrophosphate synthase (FPPS) is a key enzyme in the biosynthesis of geranylgeranyl pyrophosphate and farnesyl pyrophosphate, both of which are essential intermediates for the prenylation of small GTP-binding proteins involved in various cellular processes (19). Studies have shown that suppression of key enzymes in the mevalonate pathway, including HMG-CoA reductase and FPPS, significantly inhibits hyperglycemia-induced VSMC proliferation in murine aortas, thereby delaying the progression of atherosclerosis (19, 20). In addition, FPPS inhibition has been found to prevent norepinephrine-induced VSMC fibrosis in spontaneously hypertensive rats (21), further highlighting its role in vascular pathology. Given these findings, we hypothesize that FPPS inhibition may suppress vascular remodeling driven by VSMC proliferation. However, its specific involvement in restenosis following vascular injury and the underlying mechanisms remain unclear. To address this, we established a rat model of carotid artery injury–induced restenosis to investigate the relationship between FPPS expression and restenosis, which may provide a potential therapeutic target for its prevention and treatment.

## Experimental procedures

### Experimental animals

Pathogen-free male Sprague–Dawley (SD) rats, aged either 8 or 12 weeks, were used in this study. The animals were obtained from the Biomedicine Department of the Nanjing Institute and housed at the Medical Sciences Institute of the Zhejiang Academy under standard laboratory conditions with a regular rat chow diet. All experimental procedures were conducted in accordance with the Guide for the Care and Use of Laboratory Animals issued by the US National Institutes of Health (publication no.: 85-23, revised 1996) and were approved by the Animal Care Committee of the Medical Sciences Institute of the Zhejiang Academy, Zhejiang Province, China.

### Culturing and treatment of primary VSMCs

Primary VSMCs were isolated from the thoracic aortas of 6-week-old male SD rats using a previously established collagenase digestion method (22, 23). Cells at passages 3, 4, or 5 were used for subsequent experiments. To induce quiescence, VSMCs were first cultured in Dulbecco’s modified Eagle’s medium (DMEM; Gibco, Life Technologies) supplemented with 10% fetal bovine serum (FBS; Gibco, Life Technologies) for 24 h, followed by incubation in DMEM containing 0.1% FBS for an additional 48 h. All cell cultures were maintained under humidified conditions at 37 °C with 5% CO_2_ (3111; Thermo Scientific).

### Cell migration assay

The bottom of the culture plate was evenly scratched using a sterile 200 μl pipette tip, followed by three washes with PBS (Gibco, Life Technologies) to remove detached cells. Fresh serum-free DMEM containing transforming growth factor-beta 1 (TGF-β1, 10 ng/ml; Sigma) or angiotensin II (Ang II, 10^-7^ mol/l; Abcam) was then added, and the cells were incubated for 12 h. Cell migration was assessed by measuring the distance migrated from the scratch line using a microscope (IX73; Olympus). Five random fields of view were selected for analysis, and the longest migration distance observed was recorded as A. The rate of inhibition of cell migration was calculated using the following formula: cell migration inhibition rate (%) = (A value of control group - A value of experimental group)/A value of control group.

### Vascular restenosis after induced carotid artery balloon injury in rats

The SD rats underwent an 8-h fasting period prior to surgery. Anesthesia was induced using 3% sodium pentobarbital (30 mg/kg; Sigma), and the rats were then positioned on a sterile surgical table. After careful removal of the neck hair, the area was thoroughly disinfected. A midneck incision was made extending from the lower border of the thyroid down to the manubrium. The common carotid artery, external carotid artery, internal carotid artery, vagus nerve, and sympathetic nerves were carefully dissected following exposure of the trachea. The lower segment of the common carotid artery was temporarily clamped using an arterial clip approximately 2 cm from the bifurcation. Two thin sutures were passed under the external carotid artery: one was ligated at the distal end of the artery, and the other was positioned near the bifurcation. The internal carotid artery was then ligated using live ligation. At the lower end of the external carotid artery ligation, a small oblique incision (approximately 3–5 mm from the bifurcation) was made using ophthalmic scissors. A 1.25 mm balloon catheter (embolectomy catheter, Fogarty 2F; Edwards Laboratories) was inserted through the incision, with the balloon positioned below the bifurcation. The balloon was inflated to two atmospheric pressures (atm) and moved back and forth three times to induce damage to the intima. Afterward, the suture prepared at the bifurcation of the external carotid artery was tied. The arterial clip on the external carotid artery and the slipknot on the internal carotid artery were then removed to restore blood flow from the common carotid artery to the internal carotid artery. Finally, the muscle and skin layers were sutured, and the incision was sterilized with iodophor. Throughout the procedure, the rats' vital signs and carotid artery pulse were continuously monitored. Rats in the Sham group underwent the same surgical procedure as those in the experimental group, except no balloon injury was applied.

### Lentiviral transfection

Lentiviral transfections included both *in vitro* VSMC transfections and *in vivo* carotid arterial local transfections. The construction of the shRNA-FPPS lentiviral expression vector was performed by Jikai Gene Company. The target RNA was FPPS-RNAi (28537-2), and the interfering lentiviral vector used was GV115 (hU6-MCS-CMV-EGFP), with the target sequence GACAGCTTTCTACTCTTTC, and the GFP target sequence was ATGGTGAGCAAGGGCGAGGAGCTGTTCACCGGGGTGGTGCCCATCCTGGTCGAGCTGGACGGCGACGTAAACGGCCACAAGTTCAGCGTGTCCGGCGAGGGCGAGGGCGATGCCACCTACGGCAAGCTGACCCTGAAGTTCATCTGCACCACCGGCAAGCTGCCCGTGCCCTGGCCCACCCTCGTGACCACCCTGACCTACGGCGTGCAGTGCTTCAGCCGCTACCCCGACCACATGAAGCAGCACGACTTCTTCAAGTCCGCCATGCCCGAAGGCTACGTCCAGGAGCGCACCATCTTCTTCAAGGACGACGGCAACTACAAGACCCGCGCCGAGGTGAAGTTCGAGGGCGACACCCTGGTGAACCGCATCGAGCTGAAGGGCATCGACTTCAAGGAGGACGGCAACATCCTGGGGCACAAGCTGGAGTACAACTACAACAGCCACAACGTCTATATCATGGCCGACAAGCAGAAGAACGGCATCAAGGTGAACTTCAAGATCCGCCACAACATCGAGGACGGCAGCGTGCAGCTCGCCGACCACTACCAGCAGAACACCCCCATCGGCGACGGCCCCGTGCTGCTGCCCGACAACCACTACCTGAGCACCCAGTCCGCCCTGAGCAAAGACCCCAACGAGAAGCGCGATCACATGGTCCTGCTGGAGTTCGTGACCGCCGCCGGGATCACTCTCGGCATGGACGAGCTGTACAAGTAA. For VSMC transfections, 2 ml of primary VSMCs (5 × 10^4^ cells/ml) were seeded into a 6-well plate and incubated at 37 °C with 5% CO_2_ for 24 h. After discarding the medium, the cells were washed three times with PBS and cultured in fresh low-glucose DMEM supplemented with 10% FBS. Lentiviral solutions with various multiplicities of infection (MOI) values (20, 50, and 100) were then added to the cells for 24, 48, or 72 h. Immunofluorescence was performed to assess successful transfection, and the efficiency of FPPS knockdown was evaluated using quantitative RT–polymerase chain reaction and Western blot analysis. For *in vivo* carotid arterial local transfections, the carotid arteries from the Lenti-GFP and Lenti-FPPS shRNA groups were incubated with lentiviruses for 60 min in the vessel lumen during the induced injuries. Blood flow was temporarily obstructed at the distal ends of the internal and external carotid arteries as well as at the proximal end of the left common carotid artery during the incubation period.

### Quantitative real-time RT–PCR

Total RNA was extracted from VSMCs and carotid arteries using TRIzol reagent (Link Biotech Co, Ltd). RT–PCR was performed using the following primers: β-actin, forward 5′-CCCATCTATGAGGGTTACGC-3′ and reverse 5′-TTTAATGTCACGCACGATTTC-3′; FPPS, forward 5′-GGAGAAGGAACACGCTAATGC-3′ and reverse 5′-TGCTGAGGAGTGGCTCGTAG-3′; and connective tissue growth factor (CTGF), forward 5′-CCGCCAACCGCAAGATT-3′ and reverse 5′-TCGGGAAGGGGCAGTCA-3′. Reactions were carried out in a real-time PCR thermocycler (IQ5 Real-Time PCR Cycler; Bio-Rad), using SYBR Green (ChamQ SYBR Color qPCR Master Mix; Vazyme Biotech Co, Ltd) as the fluorescence dye. The relative expression levels were analyzed using the 2-^△△CT^ method.

### Histopathology

Rat carotid arteries were excised promptly, rinsed with prechilled normal saline, and then fixed in 4% paraformaldehyde for 24 h. After fixation, the arteries were sectioned into 5-μm slices and embedded in paraffin. H&E staining was performed to highlight the vascular intima and media structures, whereas Masson’s trichrome staining was used to assess collagen fibrosis. After staining, the samples were examined under a microscope. For frozen sections, fresh carotid tissue was embedded in optimal cutting temperature compound. The tissue was then sliced into 5-μm sections using a Cryostat slicer (RM2235; Thermo), and a fluorescence microscope was employed to assess fluorescence levels.

Formaldehyde-fixed paraffin sections from rat arteries were incubated overnight at 4°C with primary antibodies against alpha-smooth muscle actin (α-SMA, 1:200 dilution; BOSTER Biological Technology Co, Ltd; catalog no.: BM0002). The negative control lacked primary antibodies or contained goat nonimmune IgG/rabbit nonimmune IgG/secondary antibodies. In all cases, the negative control showed no significant staining. Histological and immunohistochemical analyses were performed using Image-Pro Plus 5.0 software (Media Cybernetics). The staining intensity was scored as follows: no staining (0), slight staining (1), moderate staining (2), and severe staining (3). The results are expressed as mean ± SD.

### Western blot

Western blot analysis was performed as previously described (24). Protein concentration was determined by the Bradford method (25). Samples were denatured, subjected to the SDS-PAGE using a 10% running gel, and transferred to polyvinylidene difluoride membranes (GE Healthcare), following blocking for 1 h at room temperature in 5% bovine serum albumin or 5% skim milk in Tris-buffered saline with Tween-20. The membranes were incubated overnight with the primary antibodies as follows: FPPS (Abcam; catalog no.: ab153805, 1:2000 dilution), CTGF (Abcam; catalog no.: ab6992, 1:1000 dilution), GFP (Abcam; catalog no.: ab183734, 1:1000 dilution), extracellular signal–regulated kinase (ERK; BOSTER Biological Technology Co, Ltd; catalog no.: BM4326, 1:1000 dilution), phospho-ERK (p-ERK, Cell Signaling Technology; catalog no.: 4370s, 1:1000 dilution), P38 (BOSTER Biological Technology Co, Ltd; catalog no.: BM4439, 1:1000 dilution), phospho-P38 (p-P38, Cell Signaling Technology; catalog no.: 4511s, 1:1000 dilution), Jun NH2-terminal kinase (JNK, BOSTER Biological Technology Co, Ltd; catalog no.: BM1219, 1:1000 dilution), phospho-JNK (p-JNK, Cell Signaling Technology, catalog no.: 9255s, 1:1000 dilution), β-actin (Santa Cruz; catalog no.: sc-47778, 1:1000 dilution), and GAPDH (MULTISCIENCES; catalog no.: Mab5465-100, 1:3000 dilution). Membranes were subsequently washed and incubated for an additional hour with the secondary antibody named anti-IgG horseradish peroxidase–conjugated (Bioworld; catalog no.: BS12478, 1:5000 dilution). The regions containing proteins were visualized by the enhanced chemiluminescence system (ECL Prime Western Blotting Detection Reagent; GE Healthcare). The Western blot band intensities were analyzed using Image-Pro Plus 5.0 analysis software (Media Cybernetics). The levels of the target proteins were normalized to β-actin or GAPDH, which served as internal controls, and phospho-specific proteins were normalized to the corresponding total proteins.

### Small G protein activation assay

The activity of small G proteins (Ras, RhoA, and Rac) was assessed using two methods: commercially available GTPase immunosorbent assay (GLISA) kits and laser confocal microscopy. For the GLISA assay, GLISA kits for Rac1 (catalog no.: BK128), Ras (catalog no.: BK131), and RhoA (catalog no.: BK124) were used according to the manufacturer’s instructions. Samples were lysed at the indicated time points with cell lysis buffer containing a protease inhibitor cocktail (Beyotime Biotechnology). The lysates were quantified using the Precision Red Protein Assay Reagent by measuring absorbance at 600 nm on a Versamax Plate Reader. The lysates were then diluted to 0.5 mg/ml with lysis buffer and loaded onto the GLISA plates for protein analysis. Results were obtained as absorbance values at 490 nm and were converted to total protein levels using a standard curve generated for each test.

For the laser confocal microscopy method, which was adapted from Tao *et al.* (26) with minor modifications, cells were fixed with methanol (BBI Life Sciences) at −20 °C for 15 min, followed by incubation with 0.5% Triton X-100 (BBI Life Sciences) at room temperature for 10 min. The samples were blocked with 5% bovine serum albumin (Sigma) at room temperature for 60 min. Primary antibodies (Santa Cruz) were incubated with the samples for 24 h, followed by incubation with fluorescently labeled secondary antibodies (Alexa Fluor-594; Thermo) in the dark at room temperature for 1 h. The cell nuclei were stained with 4′,6-diamidino-2-phenylindole (Sigma) at room temperature for 8 min. Fluorescence distribution was observed under a confocal laser microscope (Zeiss).

### Statistical analysis

Data are presented as mean ± SD. The significance of differences among multiple groups was assessed using one-way ANOVA with a Tukey–Kramer post hoc test. For comparisons between two groups, an unpaired two-tailed Student’s *t* test or a Mann–Whitney *U* test was used for normally distributed or non-normally distributed variables, respectively. A *p* value of <0.05 was considered statistically significant. All statistical analyses were performed using SPSS, version 22 (IBM SPSS Statistics).

## Results

### FPPS and remodeling factors of VSMCs significantly increased after stimulation with vascular-damaging reagents

**Figure 1:**
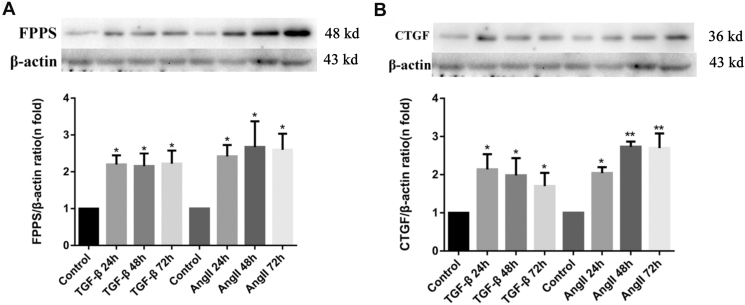
**FPPS and CTGF expression in VSMCs after stimulation with Ang II or TGF-β1.***A*, representative Western blot and quantification of FPPS protein levels in VSMCs treated with TGF-β1 (10 ng/ml) or Ang II (10^-7^ mol/l) for 24 h, 48 h, or 72 h. *B*, representative Western blot and quantification of CTGF protein levels in VSMCs under the same treatment conditions. β-actin served as the internal control. The expression levels of FPPS and CTGF were normalized to β-actin and presented as fold change relative to the control group. The proteins of FPPS, CTGF, and β-actin were from the same protein electrophoresis gel; FPPS and CTGF, along with their quantitative statistical analysis, are displayed on the *left* and *right sides* in parallel, corresponding to the same β-actin band, in order to present the figure more intuitively. Data are presented as mean ± SD from three independent experiments. Statistical significance was assessed by one-way ANOVA followed by Tukey’s post hoc test. ∗*p* < 0.05; ∗∗*p* < 0.01 *versus* control group. Ang II, angiotensin II; CTGF, connective tissue growth factor; FPPS, farnesyl pyrophosphate synthase; TGF-β1, transforming growth factor-beta 1; VSMC, vascular smooth muscle cell.

Ang II and TGF-β1 are key factors known to stimulate VSMC fibrosis (22, 27), making them suitable for this study. The optimal stimulation duration for either Ang II or TGF-β1 was first determined. Quiescent VSMCs were randomly divided into two groups for each stimulus: control and treatment (Ang II [10^-7^ mol/l] or TGF-β1 [10 ng/ml]). Each group was incubated for 24 h, 48 h, or 72 h. FPPS and CTGF protein levels were assessed by Western blotting (Fig. 1). Both proteins showed a significant increase after stimulation with Ang II or TGF-β1 for 24 h, 48 h, and 72 h, with no significant difference observed across the incubation periods (Fig. 1, *A* and *B*).

### FPPS possibly participated in fibrosis *via* regulating RhoA activity and p38 and JNK signaling pathways in VSMCs

**Figure 2:**
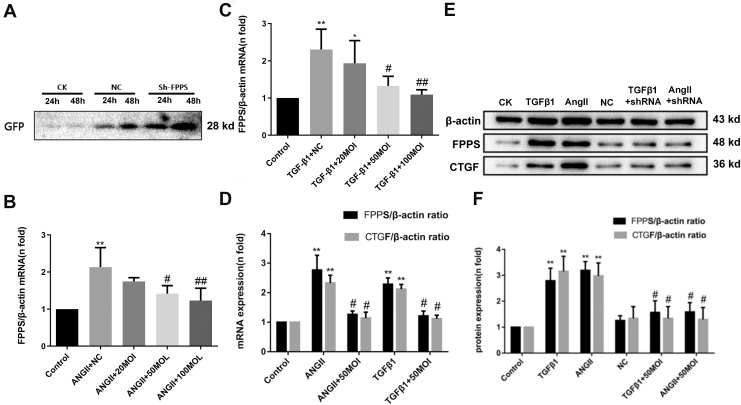
**Inhibition of FPPS by Lenti-FPPS shRNA suppresses CTGF expression at the mRNA and protein levels in VSMCs stimulated with Ang II or TGF-β1.***A*, representative Western blot of GFP expression after transfection with either empty lentivirus or Lenti-FPPS shRNA (multiplicity of infection [MOI] = 50) for 24 h and 48 h. *B* and *C*, quantification of FPPS mRNA expression following lentiviral transfection at various MOIs (20, 50, and 100) in VSMCs stimulated with Ang II (*B*) or TGF-β1 (*C*). *D*, RT–quantitative PCR analysis of FPPS and CTGF mRNA levels after pretreatment with Lenti-FPPS shRNA (MOI = 50, 48 h) followed by stimulation with Ang II or TGF-β1 for 24 h. *E*, representative Western blot of FPPS and CTGF protein expression under the same treatment conditions. *F*, quantitative analysis of protein expression, normalized to β-actin, presented as fold change relative to the control group. Data are presented as mean ± SD from three independent experiments. Statistical significance was evaluated by one-way ANOVA with Tukey’s *post hoc* test. ∗*p* < 0.05; ∗∗*p* < 0.01 *versus* control group; ^#^*p* < 0.05; ^##^*p* < 0.01 *versus* Ang II or TGF-β1 group. Ang II, angiotensin II; CK, control check; FPPS, farnesyl pyrophosphate synthase; MOI, multiplicity of infection; NC, normal control; TGF-β1, transforming growth factor-beta 1; VSMC, vascular smooth muscle cell.

We previously demonstrated that FPPS inhibitors reduced CTGF production induced by Ang II after 24 h of VSMC incubation (27). To further investigate the role of FPPS in fibrosis, lentiviral knockdowns of FPPS expression were performed in VSMCs. The virus infection conditions, including the optimal MOI and infection duration, were first determined. VSMCs were then transfected with viruses containing either empty or target genes at an MOI of 50 for 24 h and 48 h. The expression levels of GFP were assessed by Western blotting. GFP expression significantly increased in both the empty vector and sh-FPPS transfection groups, with the most pronounced expression observed at 48 h (Fig. 2*A*).

Thus, a 48-h transfection duration was chosen to detect mRNA expression of FPPS in VSMCs that had been stimulated with either Ang II (10^-7^ mol/l) or TGF-β1 (10 ng/ml) for 24 h. VSMCs were divided into the following groups: control, Ang II (TGF-β1) + NC (empty virus), Ang II (TGF-β1) + 20 MOI sh-FPPS, Ang II (TGF-β1) + 50 MOI sh-FPPS, and Ang II (TGF-β1) + 100 MOI sh-FPPS. The virus transfection groups were pretransfected with viruses for 48 h before stimulation with Ang II or TGF-β1. As shown in Figure 2, mRNA expression of FPPS significantly increased in VSMCs after 24 h of stimulation with Ang II or TGF-β1 (Fig. 2, *B* and *C*). These elevated levels were mitigated by pretransfection with 50 MOI or 100 MOI sh-FPPS but not with 20 MOI sh-FPPS. Based on these mRNA expression results, the 50 MOI sh-FPPS condition was selected for confirming the expression of FPPS and CTGF proteins. The levels of FPPS and CTGF proteins in the Ang II (TGF-β1) and Ang II (TGF-β1) + NC groups were significantly higher than those in the control group, whereas there was no significant difference between the levels in the Ang II (TGF-β1) + 50 MOI sh-FPPS and control groups (Fig. 2, *E* and *F*). These results suggest that Ang II (TGF-β1)-induced FPPS in VSMCs was effectively suppressed by pretransfection with 50 MOI sh-FPPS lentivirus for 48 h. Therefore, this virus transfection condition was selected for subsequent VSMC experiments.

**Figure 3:**
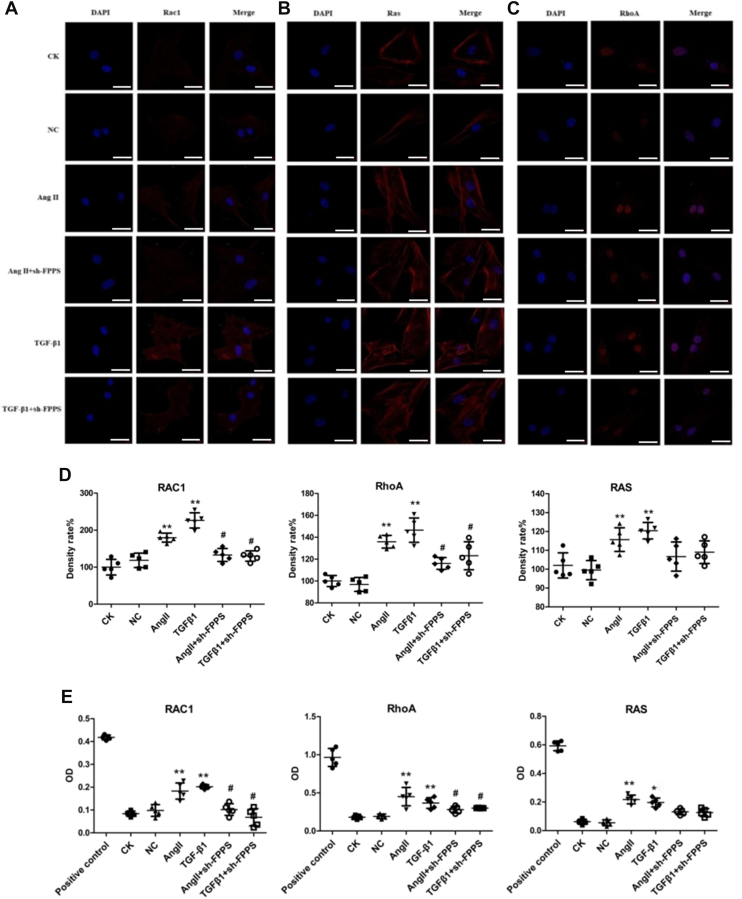
**Lenti-FPPS shRNA-mediated inhibition of FPPS attenuates small G protein activity in VSMCs stimulated with Ang II or TGF-β1.***A*–*C*, confocal immunofluorescence staining showing the expression and cellular localization of Rac1 (*A*), Ras (*B*), and RhoA (*C*) (*red*) in VSMCs across different treatment groups, with DAPI (*blue*) used for nuclear counterstaining. The scale bar represents 50 μm. *D*, quantitative fluorescence intensity analysis of Rac1, RhoA, and Ras expression based on immunofluorescence images. *E*, enzyme-linked GTPase immunosorbent assay (GLISA) analysis of Rac1, RhoA, and Ras activity under the same conditions. CK indicates untreated control cells; NC, normal control lentiviral transfection; Ang II, cells treated with angiotensin II (10^−7^ mol/l); TGF-β1, cells treated with transforming growth factor-β1 (10 ng/ml); Ang II + sh-FPPS or TGF-β1 + sh-FPPS, cells pretreated with Lenti-FPPS shRNA (MOI = 50, 48 h) before stimulation. Data are expressed as mean ± SD from five independent experiments. Statistical analysis was performed using one-way ANOVA followed by Tukey’s post hoc test. ∗*p* < 0.05, ∗∗*p* < 0.01 *versus* NC group; ^#^*p* < 0.05 *versus* Ang II or TGF-β1 group. Ang II, angiotensin II; CK, control check; DAPI, 4′,6-diamidino-2-phenylindole; FPPS, farnesyl pyrophosphate synthase; TGF-β1, transforming growth factor-beta 1; VSMC, vascular smooth muscle cell.

Next, the possible mechanisms underlying the inhibitory effect of vascular damage factors (Ang II and TGF-β1)–induced CTGF expression by sh-FPPS lentivirus transfections were investigated in VSMCs. Two methods (GLISA and laser confocal microscopy) were employed to assess the activity of small G proteins (RhoA, Ras, and Rac). Laser confocal microscopy observations revealed that the activities of Rac, RhoA, and Ras were significantly elevated after incubation with either Ang II (10^-7^ mol/l) or TGF-β1 (10 ng/ml) for 30 min. Furthermore, the activities of RhoA and Rac were reduced following inhibition of FPPS expression *via* sh-FPPS lentivirus transfections (Fig. 3, *A*–*D*). Consistent with these findings, GLISA analysis confirmed the altered activity of small G proteins in VSMCs treated with Ang II (10^-7^ mol/l) or TGF-β1 (10 ng/ml) for 30 min (Fig. 3*E*).

**Figure 4:**
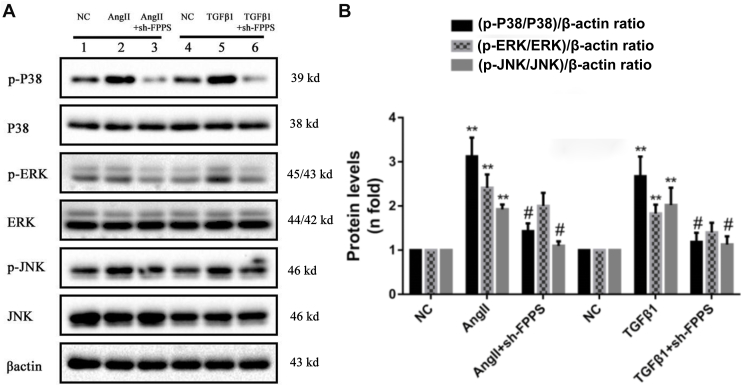
**Lenti-FPPS shRNA-mediated knockdown of FPPS attenuates MAPK pathway activation induced by Ang II or TGF-β1 in VSMCs.***A*, representative Western blots showing phosphorylation levels of p38, ERK, and JNK in VSMCs under different treatments. Total p38, ERK, JNK, and β-actin were used as internal loading controls. *B*, quantitative analysis of the phosphorylation ratios (p-p38/p38, p-ERK/ERK, and p-JNK/JNK) normalized to β-actin and expressed as fold change relative to the normal control (NC) group. VSMCs were pretreated with Lenti-FPPS shRNA (MOI = 50, 48 h) followed by stimulation with Ang II (10^-7^ mol/l) or TGF-β1 (10 ng/ml) for 24 h. Data are presented as mean ± SD from three independent experiments. Statistical analysis was performed by one-way ANOVA with Tukey’s post hoc test. ∗∗*p* < 0.01 *versus* NC group; ^#^*p* < 0.05 *versus* Ang II or TGF-β1 group. Ang II, angiotensin II; ERK, extracellular signal–regulated kinase; FPPS, farnesyl pyrophosphate synthase; JNK, Jun NH2-terminal kinase; MAPK, mitogen-activated protein kinase; MOI, multiplicity of infection; TGF-β1, transforming growth factor-beta 1; VSMC, vascular smooth muscle cell.

Finally, the involvement of the mitogen-activated protein kinase (MAPK) signaling pathway, including p-ERK1/2, p-P38, and p-JNK, was assessed to determine their role in regulating vascular injury factor–induced VSMC fibrosis *via* FPPS. As shown in Figure 4, the protein levels of p-P38, p-ERK1/2, and p-JNK in VSMCs significantly increased after incubation with Ang II (10^-7^ mol/l) or TGF-β1 (10 ng/ml) for 6 h. However, except for p-ERK1/2, all levels decreased after inhibiting FPPS expression with 50 MOI sh-FPPS lentivirus transfections (Fig. 4, *A* and *B*). These findings suggest that the P38 and JNK signaling pathways are involved in regulating VSMC fibrosis mediated by FPPS.

### FPPS involvement in vascular injury factor–induced cell migration in VSMCs

**Figure 5:**
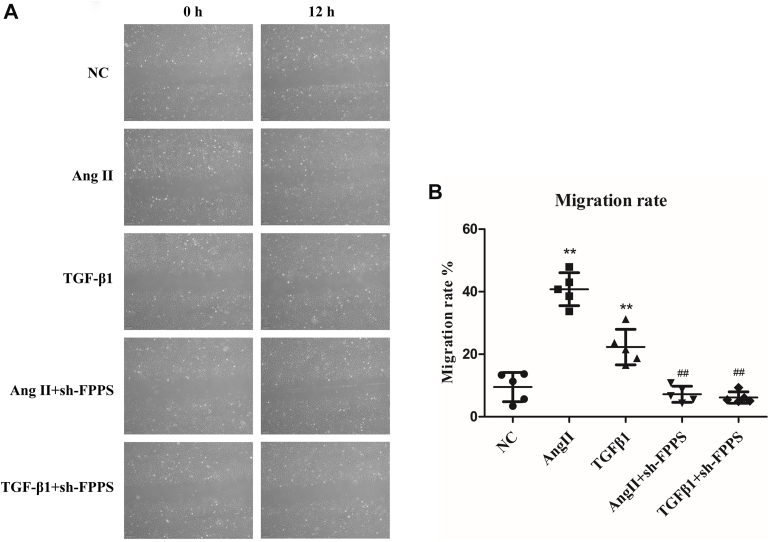
**Lenti-FPPS shRNA reduces Ang II- or TGF-β1-induced migration of VSMCs.** (A separate sh-FPPS group was not included in this study, as the primary focus was to evaluate the effect of FPPS knockdown under Ang II or TGF-β1 stimulation, simulating vascular injury conditions.) *A*, representative images of wound healing (scratch) assays at 0 h and 12 h in VSMCs under different treatments: normal control (NC), Ang II (10^-7^ mol/l), TGF-β1 (10 ng/ml), and corresponding groups pretreated with Lenti-FPPS shRNA (MOI = 50, 48 h). Images captured at 100× magnification. *B*, quantitative analysis of migration rates calculated as the percentage of wound closure area at 12 h relative to 0 h. Data are presented as mean ± SD from five independent experiments. Statistical analysis was performed using one-way ANOVA followed by Tukey’s post hoc test. ∗∗*p* < 0.01 *versus* NC group; ^##^*p* < 0.01 *versus* Ang II or TGF-β1 group. Ang II, angiotensin II; FPPS, farnesyl pyrophosphate synthase; MOI, multiplicity of infection; NC, normal control; TGF-β1, transforming growth factor-beta 1; VSMC, vascular smooth muscle cell.

Scratch assays were employed to evaluate VSMC migration, with a significant number of cells migrating to the scratch area after stimulation with Ang II or TGF-β1 for 12 h (Fig. 5*A*). However, this migration was inhibited following reduced FPPS expression because of sh-FPPS lentivirus transfections (Fig. 5*A*). The cell migration inhibition rates were statistically significant, showing 4.3 ± 13.5% in the Ang II + sh-FPPS group and 11.6 ± 6.5% in the TGF-β1 + sh-FPPS group (Fig. 5*B*). These results suggest that FPPS plays a role in VSMC migration induced by Ang II or TGF-β1.

### FPPS and CTGF levels significantly increased in the rat model of vascular restenosis

**Figure 6:**
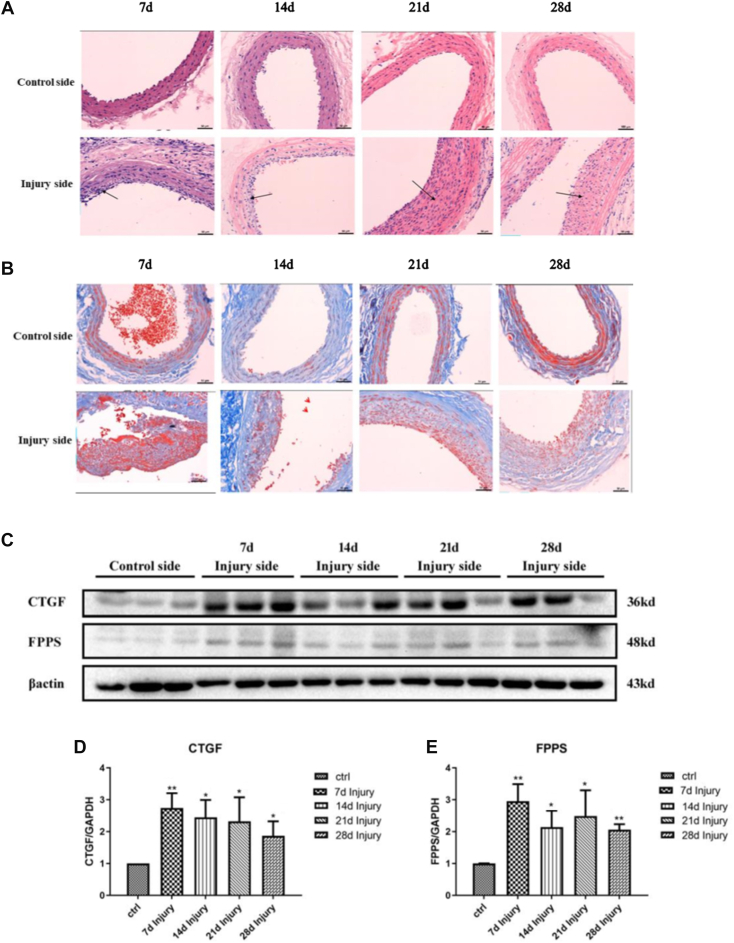
**Increased expression of FPPS and CTGF in balloon-injured carotid arteries of rats.***A*, H&E staining showing morphological changes and neointimal hyperplasia in carotid artery cross-sections at 7, 14, 21, and 28 days postinjury. *Black arrows* indicate areas of neointima formation. *B*, Masson’s trichrome staining illustrating collagen deposition (*blue*) and muscle fibers/red blood cells (*red*) in the arterial wall at corresponding time points. *C*, representative Western blot analysis of CTGF and FPPS protein expression in control and injured carotid arteries at 7, 14, 21, and 28 days. β-actin was used as a loading control. *D* and *E*, quantification of CTGF (*D*) and FPPS (*E*) protein levels normalized to β-actin and expressed as fold change relative to the control side. Data are presented as mean ± SD, and statistical analysis was performed using one-way ANOVA with Tukey’s post hoc test. ∗*p* < 0.05, ∗∗*p* < 0.01 *versus* control side. CTGF, connective tissue growth factor; FPPS, farnesyl pyrophosphate synthase.

A carotid artery balloon injury–induced vascular restenosis model was used in this study. The optimal duration for carotid artery injury leading to restenosis was first determined. SD rats were randomly assigned to five groups, each consisting of seven rats: injury for 7, 14, 21, and 28 days, with a noninjured group serving as the control. H&E and Masson staining were employed to observe the morphological changes in the vessel wall and assess the degree of fibrosis in the carotid artery, respectively. Western blotting was performed to measure the levels of FPPS and CTGF in the carotid artery. As shown in Figure 6, in the 7-day group, the intima and media of the carotid artery were necrotic and predominantly lacked cells; however, a few VSMCs had proliferated from the media into the intima, forming the neointima (Fig. 6*A*). In the 14-day group, the artery exhibited neointima with foam cells, and fibrous connective tissue had formed (Fig. 6*A*). In the 21-day group, the artery had a thickened neointima, a narrowed lumen, and more foam cells and fibrous connective tissues (Fig. 6*A*). The 28-day group showed similar results to the 21-day group (Fig. 6*A*). Masson staining revealed that the degree of collagen fibrosis in the carotid arteries increased with the duration of injury (Fig. 6*B*). Western blot analysis showed that CTGF protein levels in the carotid artery were elevated in all injury groups compared with the control, with the highest levels observed in the 7-day group (Fig. 6, *C* and *D*). FPPS levels followed a similar pattern (Fig. 6, *C* and *E*).

### FPPS involvement in restenosis after vascular injury in rats

**Figure 7:**
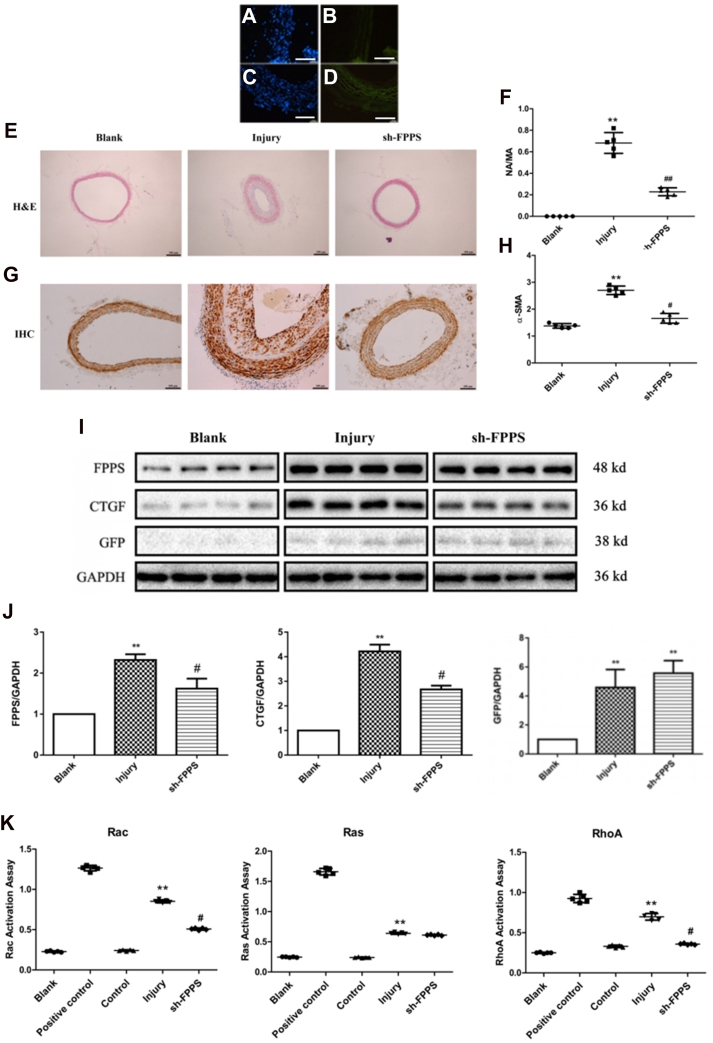
**Lenti-FPPS shRNA inhibits restenosis and reduces CTGF expression and small G protein activity in balloon-injured rat carotid arteries.***A*–*D*, immunofluorescence staining of carotid arteries in different groups: blank (no injury), injury (balloon injury without treatment), and sh-FPPS (balloon injury treated with Lenti-FPPS shRNA). Nuclei stained with DAPI (*blue*), other markers as indicated (*green*). The scale bar represents 50 μm. *E*, H&E staining showing lumen areas of carotid arteries in each group. *F*, quantitative analysis of neointimal area (NA) to medial area (MA) ratios. *G*–*H*, immunohistochemistry (IHC) staining of VSMCs for CTGF expression and corresponding quantitative analysis. *I*, representative Western blot images of FPPS, CTGF, GFP, and GAPDH in carotid arteries across groups. *J*, densitometric quantification of FPPS, CTGF, and GFP expression normalized to GAPDH, relative to the blank group. *K*, GLISA analysis of Rac, Ras, and RhoA activities in carotid arteries under different treatments. The Rac, Ras, or RhoA protein liquid contained in the commercial kit was set as the positive control group. Data are presented as mean ± SD from four to five rats per group. Statistical analysis was performed using one-way ANOVA followed by Tukey’s post hoc test. ∗∗*p* < 0.01 *versus* blank or control group; ^#^*p* < 0.05, ^##^*p* < 0.01 *versus* injury group. CTGF, connective tissue growth factor; DAPI, 4′,6-diamidino-2-phenylindole; FPPS, farnesyl pyrophosphate synthase; GLISA, GTP protease–linked immunosorbent assay; MA, media area; NA, neointimal area; VSMC, vascular smooth muscle cell.

Lentiviral knockdowns of FPPS expression were employed to assess the involvement of FPPS in restenosis following vascular injury. Thirty-six rats were randomly assigned to three groups: control group (Lentivirus with GFP, Lenti-GFP [3 × 10^8^ plaque-forming unit], n = 12), lentivirus knockdown FPPS group (Lenti-FPPS shRNA [6 × 10^8^ plaque-forming unit], n = 12), and sham group (n = 12). Carotid arteries from the Lenti-GFP and Lenti-FPPS shRNA groups were incubated with lentiviruses for 60 min in the vessel lumen during injury. For the sham group, a similar procedure was followed, except that the balloon was not used to damage the blood vessels, and no viruses were incubated. Carotid arteries were harvested 28 days postprocedure for histopathology and Western blot analysis. As shown in Figure 7, the neointima and media of the carotid arteries in the lentiviral treatment groups displayed high fluorescence, particularly in the media, whereas the sham group lacked fluorescence (Fig. 7, *A*–*D*). Western blotting confirmed that GFP expression levels in the lentiviral treatment groups were significantly higher than in the controls (Fig. 7, *I* and *J*). These results indicate that the carotid arteries were successfully transfected with lentiviruses.

Subsequently, H&E staining was performed to assess changes in vascular structure. The areas of neointima and media were measured using Image-Pro Plus 5.0 software (Media Cybernetics), and the ratio of neointimal area to medial area was used as an indicator of stenosis in the carotid arteries. Rats in the Lenti-GFP group exhibited more neointima and more severe stenosis compared with the sham group. This vascular remodeling was significantly reversed by Lenti-FPPS shRNA transfection (Fig. 7, *E* and *F*). In addition, α-SMC-actin immunohistochemistry was utilized to evaluate VSMC proliferation in the neointima. The staining intensity was calculated by subtracting the background staining from the intensity of most cells. Staining intensities were scored as follows: no staining (0), slight staining (1), moderate staining (2), and severe staining (3). Positive α-SMA staining was primarily observed in VSMC cytoplasm in the sham group and in neointimal cell cytoplasm in the control group (Fig. 7, *G* and *H*). The α-SMA positive intensity scores indicated that Lenti-FPPS shRNA transfection inhibited VSMC proliferation in the neointima (Fig. 7, *G* and *H*).

Finally, the protein levels of FPPS and CTGF, as well as the activities of small G proteins (RhoA, Ras, and Rac) in the carotid arteries, were assessed. Unlike the *in vitro* experiments, the activities of small G proteins were evaluated using the GLISA method only. As shown in Figure 7, both FPPS and CTGF protein levels increased after balloon injury for 28 days, but these levels decreased following transfection with Lenti-FPPS shRNA (Fig. 7, *I* and *J*). GLISA activation assays revealed that the activities of RhoA, Ras, and Rac all increased after balloon injury, with Rac and RhoA activity decreasing following transfection with Lenti-FPPS shRNA (Fig. 7*K*).

## Discussion

The main findings of this study are as follows: (1) The expression of FPPS and CTGF in VSMCs was significantly upregulated after stimulation with the vascular injury factors Ang II and TGF-β1. (2) The activity of small G proteins and the migration of VSMCs were enhanced under Ang II or TGF-β1 stimulation. (3) Lenti-FPPS shRNA reduced the stimulatory effects of Ang II or TGF-β1 on FPPS, CTGF, phosphorylated proteins (P38 and JNK), small G protein activities, and VSMC migration. (4) A rat model of restenosis was successfully established following balloon-induced carotid artery intimal injury. This model was characterized by a narrowed lumen, large areas of neointima in the intima and media, abundant foam cells, and fibrous connective tissue formation. (5) Restenosis, along with the increased levels of FPPS, CTGF, and small G protein activities induced by vascular injury in the carotid artery, was alleviated following treatment with Lenti-FPPS shRNA.

Restenosis following PCI remains a prominent research focus in the field of coronary interventional therapy. Vascular endothelial injury and subsequent repair can be triggered by various mechanical, hemodynamic, or infectious factors. Traditionally, it was believed that the primary response to vascular injury involves endothelial cell loss and proliferation. However, while endothelial cells share some characteristics with VSMCs, significant morphological differences remain (28). The widely accepted mechanism of restenosis involves the proliferation, migration, adhesion, and phenotypic transformation of VSMCs in response to intimal injury. This process is accompanied by ECM protein synthesis, collagen formation, and phenotypic changes, which are also linked to low-density lipoprotein endocytosis (29). In line with previous studies, the present research demonstrated that the intima and media of the carotid artery were necrotic, with cell loss and extensive proliferation and migration of VSMCs from the media into the intima. This resulted in the formation of a thickened neointima, containing foam cells and fibrous connective tissue (Fig. 6*A*). In addition, Masson staining revealed that collagen fibrosis in the carotid arteries increased progressively with the duration of injury (Fig. 6*B*). These findings confirm the successful establishment of a balloon injury–induced vascular restenosis model.

VSMCs play a crucial role in the development of vascular restenosis. Mechanical manipulation of the vascular intima can directly stimulate the proliferation and aggregation of VSMCs, ultimately leading to neointima formation, which alters the structure and function of the vascular wall (30, 31). A key pathological feature of vascular stenosis is the phenotypic transition of VSMCs. This transition is characterized by excessive proliferation, migration, and ECM secretion by VSMCs, resulting in a proliferative tissue in the local intima dominated by VSMCs and inflammatory cells (32). Thus, inhibiting or reversing this phenotypic transition is a promising strategy for treating vascular restenosis. In addition, autopsy studies of restenosis following PTCA have shown that the ECM, which forms a dense network linking cells together, accounts for 89% of the neointima (33). Collagen fibers, which are the primary components of the ECM, have been suggested as key contributors to restenosis and vascular remodeling because of their accumulation (34). CTGF has been identified as a potent profibrogenic factor in vascular remodeling, promoting VSMC proliferation and migration (35). Furthermore, CTGF is overexpressed in human atherosclerotic lesions (36) as well as in aortas and cultured VSMCs stimulated with Ang II (22). In this study, we found that both mRNA and protein levels of CTGF in VSMCs were significantly elevated after stimulation with the vascular injury factors Ang II or TGF-β1. Moreover, CTGF expression was markedly increased in the injured carotid arteries *in vivo*, coinciding with thickening of the vessel wall. In addition, the neointima contained numerous VSMCs that migrated from the media and deposited abundant collagen fibers, further supporting the involvement of CTGF in the process of vascular restenosis.

FPPS is a key enzyme in the mevalonate pathway, responsible for generating intermediates, such as farnesyl pyrophosphate and geranylgeranyl pyrophosphate, through the prenylation reaction. These intermediates regulate the activity of small G proteins, MAPK signaling pathways downstream of small G proteins, and various cellular processes, including proliferation, growth, and apoptosis (21, 37). Previous studies have highlighted the regulatory role of FPPS in different vascular injury models. For instance, in a mouse model of diabetic vascular complications, FPPS promoted VSMC proliferation by modulating small G protein activity, contributing to vascular smooth muscle fibrosis and dysfunction, which accelerated the progression of atherosclerosis (20). Another study reported that ibandronate, a potent FPPS inhibitor, prevented norepinephrine-induced VSMC fibrosis in spontaneously hypertensive rats by inhibiting FPPS activity, potentially through the inhibition of the Ras/p38 MAPK signaling pathway (21). Furthermore, we previously demonstrated that FPPS inhibitors reduced CTGF production in VSMCs after 24 h of Ang II stimulation (27). These findings suggest that FPPS plays a critical “molecular bridge” role in vascular remodeling. However, the involvement of FPPS in vascular restenosis following mechanical injury to the vascular intima remains to be explored.

The present study, for the first time, investigated the role of FPPS in restenosis following vascular injury, both *in vitro* and *in vivo*, and elucidated the underlying mechanism of FPPS in vascular stenosis. *In vitro*, the expression of FPPS and CTGF, along with the migration of primary VSMCs, was significantly upregulated after stimulation with vascular injury factors Ang II or TGF-β1. To assess whether these effects could be mitigated by interfering with FPPS expression, we employed Lenti-FPPS shRNA for FPPS inhibition. As expected, inhibition of FPPS by Lenti-FPPS shRNA resulted in decreased CTGF expression and reduced VSMC migration. The underlying mechanism was also explored. Small G proteins, classified into five families (Ras, Rho, Rab, Sar/Arf, and Ran), regulate various cellular processes, and their activities often involve cascades and crosstalk within and between these families. Post-translational modification of small G proteins, particularly isoprenylation, is critical for cellular protein–protein interactions and membrane-associated protein transport (38, 39). Prenylated small G proteins have been implicated in various cardiovascular diseases (40). Consequently, we examined the activity of small G proteins downstream of FPPS, revealing that the activities of Rac and RhoA were significantly reduced after FPPS inhibition. It is well established that MAPKs, which act downstream of small G proteins, are a crucial group of enzymes in vascular remodeling, particularly in response to Ang II stimulation (41, 42). Our results demonstrated that inhibition of FPPS by Lenti-FPPS shRNA reduced the phosphorylation of JNK and p38 MAPKs, suggesting that FPPS modulates the MAPK pathway *via* small G proteins. Compared with our previous studies that utilized small-molecule inhibitors to suppress FPPS activity, the present study employed a gene-silencing strategy *via* Lenti-FPPS shRNA, which offers higher specificity, longer-lasting inhibition, and compatibility with *in vivo* delivery. More importantly, we established a carotid artery balloon injury model to investigate FPPS-mediated vascular remodeling under pathological conditions, which had not been addressed in our earlier work. This combined approach of gene silencing and *in vivo* modeling provides a more comprehensive understanding of the molecular mechanisms underlying restenosis and reinforces the potential of FPPS as a therapeutic target. Although the use of pharmacological FPPS inhibitors could further support the mechanistic findings of this study, we did not employ systemic FPPS inhibition in our *in vivo* experiments because of concerns about potential off-target effects associated with long-term suppression. Instead, this study focused on achieving localized gene silencing through Lenti-FPPS shRNA, aiming for a more specific effect on vascular tissues and minimizing systemic side effects. Future studies may consider exploring targeted or controlled-release formulations of FPPS inhibitors to validate our findings while limiting nonspecific exposure. In parallel, an *in vivo* vascular injury restenosis model was established using balloon-induced carotid artery intimal injury. As anticipated, intimal hyperplasia and overall vascular remodeling were significantly reversed after FPPS inhibition by Lenti-FPPS shRNA. Similar to the *in vitro* findings, the activities of small G proteins, including Rac and RhoA, were reduced, further supporting the role of FPPS in regulating vascular remodeling through small G protein-mediated pathways. Although the degree of FPPS knockdown achieved *in vivo* in this study was relatively modest, previous research has shown that its downstream signaling pathways—particularly small GTPases, such as Rac, Ras, and RhoA—are highly sensitive to even slight changes in FPPS expression. Minor alterations in FPPS levels can significantly affect isoprenoid synthesis, thereby influencing the prenylation and membrane localization of small GTPases (43). Moreover, CTGF, a key profibrotic effector downstream of these pathways, has been reported to respond markedly to subtle upstream perturbations (44, 45). This cascade amplification mechanism may explain why moderate FPPS reduction led to the pronounced neointimal hyperplasia and decreased small GTPase activity observed in this study.

Despite the valuable insights provided by this study, there are some limitations to consider. First, although a restenosis model was established by balloon injury to normal carotid arteries in rats, this does not fully replicate the response of an atherosclerotic vascular wall following balloon injury. Nevertheless, this model remains the most commonly used for studying vascular restenosis. While it may not mirror the human condition precisely, it provides valuable information on cell proliferation, migration, and regulation and serves as a simple, cost-effective approach for investigating antiproliferative drugs and genes *in vivo*. Furthermore, no single animal model can completely replicate the entire process of human restenosis. Thus, the specificity and limitations of animal models must be considered when extrapolating findings to human atherosclerosis. Second, because of the small amount of vascular tissue available after lentiviral transfection, the expression of MAPK proteins in the vascular tissue could not be assessed. However, based on the results from both the cell and animal experiments, it is hypothesized that the expression of MAPK proteins in the vascular tissue following lentiviral transfection would follow a similar trend to the cell results. Future studies are necessary to confirm this hypothesis.

## Conclusion

In summary, this study elucidated the role of FPPS in regulating the proliferation, migration, phenotypic transformation, ECM synthesis, and matrix metalloproteinases/tissue inhibitors of metalloproteinases of VSMCs and the underlying molecular mechanisms *in vitro*. In addition, the study clarified the expression and functional changes of FPPS, small G proteins, and CTGF in the vascular wall during vascular remodeling *in vivo*. FPPS contributes to restenosis after vascular injury by modulating the balance of matrix metalloproteinases/tissue inhibitors of metalloproteinases through small G protein regulation. These findings highlight FPPS as a potential target enzyme for preventing and treating vascular remodeling.

## Data availability

The data that support the findings of this study are available from the corresponding author, Changqing Du, upon reasonable request.

## Declaration of generative AI and AI-assisted technologies in the writing process

During the preparation of this work, the authors did not use generative AI or AI-assisted technologies in the development of this article.

## Conflict of interest

The authors declare that they have no conflicts of interest with the contents of this article.
